# Understanding the system dynamics of obesity-related behaviours in 10- to 14-year-old adolescents in Amsterdam from a multi-actor perspective

**DOI:** 10.3389/fpubh.2023.1128316

**Published:** 2023-05-25

**Authors:** Angie Luna Pinzon, Karien Stronks, Helga Emke, Emma van den Eynde, Teatske Altenburg, S. Coosje Dijkstra, Carry M. Renders, Roel Hermans, Vincent Busch, Mai J. M. Chinapaw, Stef P. J. Kremers, Wilma Waterlander

**Affiliations:** ^1^Amsterdam UMC location University of Amsterdam, Department of Public and Occupational Health, Amsterdam, Netherlands; ^2^Amsterdam Public Health Research Institute, Health Behaviors and Chronic Diseases, Amsterdam, Netherlands; ^3^Amsterdam UMC location Vrije Universiteit Amsterdam, Department of Public and Occupational Health, Amsterdam, Netherlands; ^4^Department of Health Sciences, Faculty of Science, Vrije Universiteit Amsterdam, Amsterdam, Netherlands; ^5^Obesity Center CGG, Erasmus MC, University Medical Center Rotterdam, Rotterdam, Netherlands; ^6^Department of Health Promotion, NUTRIM School of Nutrition and Translational Research in Metabolism, Maastricht University, Maastricht, Netherlands; ^7^Sarphati Amsterdam, Public Health Service (GGD), Amsterdam, Netherlands

**Keywords:** overweight and obesity, adolescents, systems thinking, complex systems, causal loop diagram, system dynamics

## Abstract

**Introduction and Methods:**

To develop an understanding of the dynamics driving obesity-related behaviours in adolescents, we conducted systems-based analysis on a causal loop diagram (CLD) created from a multi-actor perspective, including academic researchers, adolescents and local stakeholders.

**Results:**

The CLD contained 121 factors and 31 feedback loops. We identified six subsystems with their goals: (1) interaction between adolescents and the food environment, with profit maximisation as goal, (2) interaction between adolescents and the physical activity environment, with utility maximisation of outdoor spaces as goal, (3) interaction between adolescents and the online environment, with profit maximisation from technology use as goal, (4) interaction between adolescents, parenting and the wider socioeconomic environment, with a goal focused on individual parental responsibility, (5) interaction between healthcare professionals and families, with the goal resulting in treating obesity as an isolated problem, and (6) transition from childhood to adolescence, with the goal centring around adolescents’ susceptibility to an environment that stimulates obesity-related behaviours.

**Discussion:**

Analysis showed that inclusion of the researchers’ and stakeholders’ perspectives contributed to an understanding of how the system structure of an environment works. Integration of the adolescents’ perspective enriched insights on how adolescents interact with that environment. The analysis further showed that the dynamics driving obesity-related behaviours are geared towards further reinforcing such behaviours.

## 1. Introduction

Public health problems such as childhood overweight and obesity result from the interaction of multiple factors within a complex adaptive system. A complex adaptive system can be defined as a collection of interconnected factors that is more than the sum of its parts (1). Such factors operate at multiple levels – ranging from individual behaviours like the amount of sedentary time to more upstream factors related to the economic, sociocultural, physical and political environments (2). Identifying such factors and interconnections is considered an important step in gaining an understanding of a complex adaptive system. This understanding can enable action to bring about systems change, and it can serve as a basis to assess changes over time (3, 4).

One way of developing a system understanding is through *system mapping*. A frequently used mapping tool is the *causal loop diagram* (CLD) (5–7). Such diagrams provide visual representations of the complexity of a problem, depicted in the form of factors, causal relationships, polarity and feedback loops (8). A well-known example of a CLD system map is the Foresight map. It identifies a broad range of factors that influence childhood overweight and obesity, thus providing a ‘whole’ picture of the system (9). At the core of the Foresight map is ‘energy balance’ around which are over 100 interconnected factors clustered in seven major sub-systems directly or indirectly affecting energy balance. For the first time, this map showed that obesity results from many interconnected policy, environmental, social, economic, cultural, behavioural, and biological causes. While succeeding in effectively illustrating the wide range of causes of obesity, the Foresight map was developed by experts based on empirical research literature, and it thus creates an *academic perspective* on the system in question.

Another potential important perspective to take into account is that of *stakeholders* (6). Friel et al. (10) for example conducted collaborative conceptual modelling workshops with stakeholders from different sectors in Australia including academia, non-governmental health organizations and government to create a system map that illustrated the multiple factors associated with inequities in healthy eating. This system map resulted in the identification of seven sub-systems including (1) food supply and environment; (2) transport, (3) housing and the built environment, (4) employment, (5) social protection, (6) health literacy, and (7) food preferences. One more potential important perspective to consider is that of the *targeted group* itself, often identified through methods such as group model building (GMB) (11–14). Savona et al. (14) conducted for example GMB with adolescents in five European countries in order to map the factors that they considered to be important obesity drivers. In the overall systems map that represented the perspective of more than 200 adolescents, three sub-systems stood out: (1) commercial drivers of adolescents’ unhealthy diet, (2) mental health and unhealthy diet, and (3) social media use, body image and motivation to exercise.

A common characteristic of such CLDs is that they provide a single perspective on the system – a perspective of experts based on research literature or a perspective of stakeholders or of the target group. What is still missing, to our knowledge, is a system map or CLD that *integrates* multiple perspectives, including those from experts, various stakeholder groups and the target group itself. Such a *multi-actor perspective* is important because different actors have different perceptions of the causes of a problem, and these influence the ways in which the system can be changed (15–17). Hence, when mapping a system, one should ideally consider the perspectives of the various actors in order to obtain a more complete system understanding (15, 16, 18). Indeed, in their framework for transformative systems change, Foster-Fishman and colleagues have described such a system understanding from a multi-actor perspective as a key step in the process towards effecting systems change, as this accentuates the subjective nature of understanding systems (16).

Another common characteristic of most CLD papers in the literature, including the abovementioned examples, is that these mostly focus on developing and understanding of the system in terms *of system structure*, describing the included factors, connections and feedback loops of a particular problem (5). Foster-Fishman and colleagues further emphasise in their framework that one not only needs an understanding of the *system structure* when trying to understand the targeted system, but an understanding of the *system function* is also required in order to change the status quo of a system. Such a system function understanding includes a more in-depth analysis of the system as a whole, which identifies and understands the deeper system dynamics in terms of structure, goals and paradigm (16, 19, 20).

In this paper, we aim to identify and understand the underlying system dynamics that drive obesity-related behaviours in 10- to 14-year old adolescents in Amsterdam, by conducting systems-based analysis from a multi-actor perspective. We report on how we applied systems dynamics methods to assess the extent to which these methods led to new understandings of the targeted problem in the local context.

## 2. Methods

### 2.1. The LIKE programme

The results presented in this study are part of the larger Lifestyle Innovations Based on Youth Knowledge and Experience (LIKE) programme (21), which is part of the Amsterdam Healthy Weight Programme, a local-government-led whole-systems approach (22). The LIKE programme is designed to tackle childhood overweight and obesity in 10- to 14-year-old adolescents in three neighbourhoods with a low socioeconomic status in the Amsterdam East city district in the Netherlands. It combines a system dynamics and participatory action research approach in order to develop, implement and evaluate a dynamic action programme.

To arrive at such dynamic action programme, the first part of LIKE focuses on developing an understanding of the targeted system. In LIKE, we refer to this system understanding as the pre-existing system of obesity-related behaviours in 10- to 14- year-old adolescents in Amsterdam. We allude to ‘pre-existing system’ because in systems evaluations, there is no control or baseline system, rather, the system continuously changes over time either with or without intentional intervention (3).

#### 2.1.1. Procedures

In LIKE, we combine three different perspectives to achieve a system understanding. The academic researchers’ perspective provides an external view of the system and was published here (23). In this paper we enriched our system understanding by adding the adolescents’ and stakeholders’ perspectives to provide an additional internal view of the system. On top of that, we conducted system-based analysis to understand the underlying system dynamics. This was operationalised by following a three-step process. First, data were collected using qualitative methods separately from the different perspectives. The data were then integrated to arrive at an overarching map, or CLD, of the pre-existing system. Finally, the resulting CLD was analysed using system-based methods to qualitatively understand the underlying system dynamics. The exact procedures are detailed below. Ethical approval for the data collections was obtained from the institutional medical ethics committee of Amsterdam UMC, Location VUMC (2018.234).

### 2.2. Step 1. Data collection from a multi-actor perspective

To operationalise the central aim of identifying and understanding the underlying system dynamics that drive obesity-related behaviours we focused on four behaviours that are particularly significant to childhood overweight and obesity and which are also the focus of the Amsterdam Healthy Weight Programme. These include dietary behaviour, physical activity, sedentary behaviour and sleep. We conducted an in-depth needs assessment in LIKE between 2018 and 2021 to gain insights of the system dynamics that related to these four behaviours. Of note, as our focus was in uncovering the system dynamics, we collected data that accounted for the change over time of factors influencing the four targeted behaviours, rather than a static situation. A central question for the collection of data was therefore: “What factors explain the dynamics in dietary behaviour, physical activity, sleep, and sedentary behaviour, in 10- to 14- year-old adolescents Amsterdam in the past three decades?.” During the needs assessment period, various qualitative methods were employed, including the construction of CLDs by academic researchers based on research literature (23); construction of CLDs by adolescents (Emke et al., unpublished data, 2022); GMB with stakeholders, including parents and other actors in the direct environment of adolescents (schoolteachers, sport coaches etc.) (Waterlander et al., unpublished data, 2022); and interviews with healthcare professionals (HCPs) (24), (Van den Eynde et al., 2022).

#### 2.2.1. Researchers’ perspective

As mentioned above, the academic researchers’ perspective on the pre-existing system had previously been captured in LIKE during 2019–2020 (23). First, factors were retrieved from systematic reviews (*n = 190 factors*). Next, factors were connected by taking into account their causal relationship. A positive polarity marked positive causation meaning that as a cause increases, the effect also increases; or that as a cause decreases, the effect also decreases (more chicken leads to more eggs). A negative polarity marked inverse causation meaning that as a cause increases, the effect decreases; or that as a cause decreases, the effect increases (more foxes leads to less chicken) (23). A total of four CLDs were created around physical activity (*n* = 20 factors), dietary behaviour (*n* = 28 factors), sedentary behaviour (*n* = 19 factors) and sleep (*n* = 13 factors). These CLDs revealed the presence of dynamics including feedback loops, mechanisms and subsystems. Highlighted subsystems included for example home and school environments but also newly identified subsystems such as urban systems, social welfare and macroeconomics. For more details on the construction of these four CLDs and results hereof, we refer to the work of Waterlander and colleagues (23).

#### 2.2.2. Adolescents’ perspective

Participatory action groups were conducted between 2018 to 2020 at two primary and two secondary schools located in the LIKE target areas in Amsterdam East. Participatory action groups consisted of four to eight adolescents aged 10 to 14 and an academic facilitator. In these participatory groups, adolescents were first trained in research skills, and they subsequently investigated, among their peers, the factors that influenced their dietary behaviour, physical activity, sedentary behaviour and sleep. Adolescents then analysed the collected data separately for primary and secondary schools and summarised the major factors *(n = 126 factors)* associated with the four targeted behaviours into six CLDs (three constructed by primary school children and three CLDs by secondary school adolescents). From these CLDs, three overarching subsystems were identified: (1) Adolescents live in a physical activity environment with easy access to unhealthy food products, (2) Social norm around unhealthy behaviours are formed by peers, friends and family, and (3) Unhealthy behaviours are interrelated and reinforce each other. Details of the participatory action group process will be published elsewhere (Emke et al., unpublished data, 2022).

#### 2.2.3. Stakeholders’ perspective

The stakeholders’ perspective was captured through two different methods. First, four GMB workshops were held in 2020–2021 in Amsterdam East. 29 to 31 stakeholders participated in the different rounds and represented the sectors schools, healthcare, local government, the Amsterdam Healthy Weight Programme, sports clubs, and community and youth organisations (including volunteers and parents). During the GMB workshops, participants constructed a CLD around dietary behaviour, physical activity, sedentary behaviour and sleep *(n = 39 factors)*, in adolescents from their perspective as local stakeholders. This CLD revealed the presence of five subsystems: (1) the food environment, (2) the home environment, (3) sleep, (4) physical activity, and (5) transition from 10 to 14 years. The details of the GMB process will be part of a separate paper (Waterlander et al., unpublished data, 2022).

Lastly, interviews with 18 HCPs were conducted in 2019–2020 to gather data about barriers and facilitators that bear upon obesity-related behaviours in adolescents with obesity and their parents. These barriers and facilitators were summarised into seven themes including (1) individual child factors, (2) role of the parents, (3) physical environment, (4) socioeconomic environment, (5) cultural environment, (6) family’s experience with healthcare, and (7) family’s motivation. For more details on these results we refer to (24). Moreover, the HCPs interviews data were also used to identify barriers and facilitators that influence the professional support and care for adolescents with obesity and their parents. Identified themes included for example conducting a biomedical, psychosocial and lifestyle assessment, tailoring the approach to the adolescent and parents’ needs, and investing in building a relationship. Details will be provided elsewhere (Van den Eynde et al., 2022).

### 2.3. Step 2. Developing the map of the pre-existing system

On the basis of the data sources outlined above, the next step involved the integration of the data to arrive at a multi-actor perspective CLD of the pre-existing system. The process is outlined below. Maps were first created using STICK-E software (STICK-E version 3, Deakin University) and then imported in KUMU (Relationship mapping software, 2022) for editing purposes. The final representation of the pre-existing map was edited in Adobe Illustrator CS5.

#### 2.3.1. Step 2.1. Merging the researchers’ literature-based CLDs

The first step consisted of constructing a ‘baseline’ CLD system map. As input for this baseline CLD, the four separate CLDs (consisting of factors and their interconnections) – relating to adolescents’ dietary behaviour, physical activity, sedentary behaviour and sleep, representing the academic perspective (23) – were merged into an overarching baseline CLD covering all four behaviours. System map development started with the researchers’ perspective because those CLDs were already published while the CLDs from the other perspectives were still being developed. Integration of the four separate CLDs was performed by merging the CLDs on the basis of common factors. For example, the sedentary behaviour CLD was linked with the sleep CLD by the factor ‘screen use’, which was present in both CLDs. Next, the resulting baseline CLD was iteratively refined by removing duplicate variables and by making sure each factor was at the same level of detail and specificity (25). For example, the factors ‘screen use for school or work’ and ‘use of screen-based social media by adults’ were incorporated into the ‘screen use as social norm’ factor. This process resulted in a baseline system map that reflected the researchers’ perspective.

#### 2.3.2. Step 2.2. Adding the adolescents’ perspective

The next step involved integrating the perspective of adolescents into the baseline map. Factors associated with dietary behaviour, physical activity, sedentary behaviour and sleep that were present in the six CLDs constructed by adolescents (Emke et al., unpublished data, 2022), but still absent in our evolving map, were extracted. Examples include ‘gaming’, ‘nightmares’, ‘biking’, and ‘supermarket proximity’. As well single factors as connections between the factors were added to the map. These connections were based upon the causal connections and polarity identified by adolescents in the original six CLDs.

#### 2.3.3. Step 2.3. Adding the stakeholders’ perspective

Integration of the stakeholders’ perspective into the system map followed a two-step process. First, factors present in the stakeholders’ CLD (produced in the GMB workshops) but still absent in our system map were added. Those factors related to issues such as health (e.g., ‘listening to your own body’, ‘health as a priority’) and the home environment (e.g., ‘parents as role models’, ‘parents in survival mode’). Connections between the newly added factors were drawn by the present authors reflecting the direction of causality between factors as observed in the original stakeholders’ CLD. Second, the interview data from HCPs were incorporated. As previously mentioned, these data were used to identify themes around barriers and facilitators influencing both obesity-related behaviours in adolescents with obesity and their parents (24) as well as around the professional support and care that those adolescents and parents receive (Van den Eynde et al., 2022). Because those data were not in the form of CLDs, we reviewed the identified themes and sub-themes and treated these as factors in order to add these to our system map. Examples of newly added factors include ‘parents being supportive and involved’ and ‘vagueness of the healthcare system’ (24). Some factors from the original data were not added, because their level of detail and specificity did not equate with that of the factors already included (overly broad formulations such as ‘obesogenic environment’ or ‘the healthy choice should be the easy choice’). Because the original HCPs data merely noted factors and made no connections between them, we iteratively drew connections and identified directions of causality, based on our interpretations of the data. The researcher that collected the original data (EvdE) closely monitored this process.

#### 2.3.4. Step 2.4. Identification of feedback loops

Lastly, the connections and directions of causality between all factors in the evolving system map were re-assessed to facilitate identification of feedback loops. A feedback loop refers to a sequence of factors and interconnections that creates a closed loop of causal influences (3). Feedback loops can either be reinforcing, which indicates exponential growth or decay, or balancing, indicating stabilisation or tending to equilibrium (26). The identification of reinforcing and balancing feedback loops was performed by ALP and WW, and reviewed by the rest of authors of the present study. Altogether, this process resulted in the creation of a multi-actor map of the pre-existing system of obesity-related behaviours in adolescents.

### 2.4. Step 3. System-based analysis of the map of the pre-existing system

In the final step, a system-based analysis (22) of the CLD of the pre-existing system was performed to gain an understanding of the dynamics of obesity-related behaviours. This analysis was performed using the Intervention Level Framework developed by Johnston and colleagues and is based on five levels: system paradigm, goals, structure, feedback loops and elements (27). We used the Intervention Level Framework to distinguish the structure and function of the pre-existing system.

To understand the system structure, we analysed the CLD to assess the identified factors (ILF level *elements*) and feedback loops (ILF level *feedback loops*). The clustering of feedback loops revealed the presence of specific themes that helped us identify subsystems and the overall system structure (ILF level *structure*). The identification of the system structure as well as the subsystems was carried out iteratively though group discussions by the authors until consensus was reached. To understand the system function, we subsequently tried to identify subsystem goals (ILF level *goals*) and the overarching system paradigm (ILF level *system paradigm*). This was done by building on existing expert knowledge on system function, for example as detailed in the report of the Lancet Commission on Obesity (28). Finally, both the map of the pre-existing system and the system-based analysis were reviewed by all authors to make sure all collected data were accurately presented in the CLD and correctly interpreted.

## 3. Results

**Figure 1:**
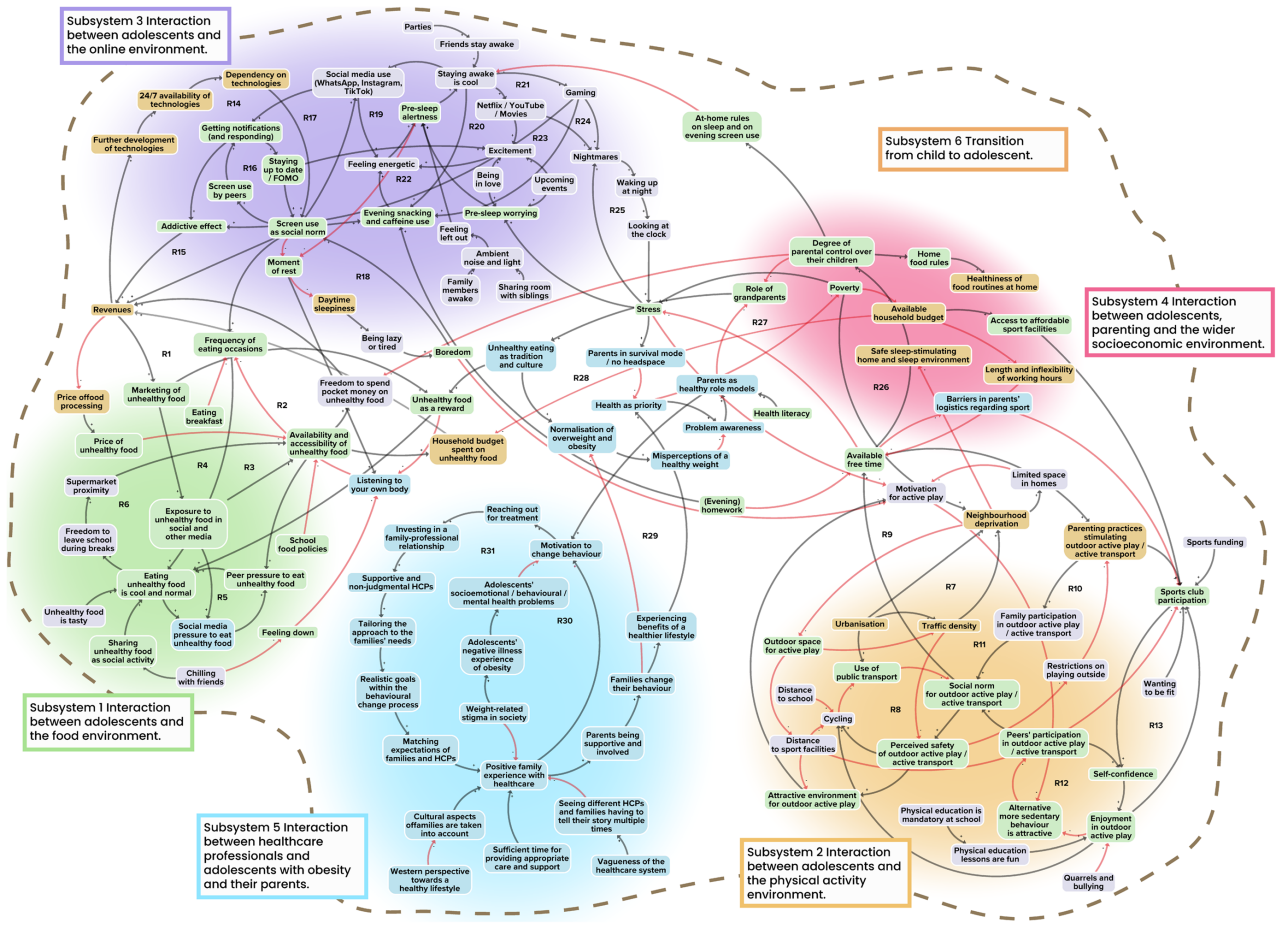
Pre-existing system of obesity-related behaviours in an integrated multi-actor perspective with identified subsystems. Factors derived from the researchers’ perspective are shown in yellow, those from the adolescents’ perspective in purple, and those from the stakeholders’ perspective in blue. Factors present in at least two of the three perspectives are shown in green. Black arrows indicate positive polarity and red arrows indicate negative polarity in the causal relationship between factors.

**Figure 2:**
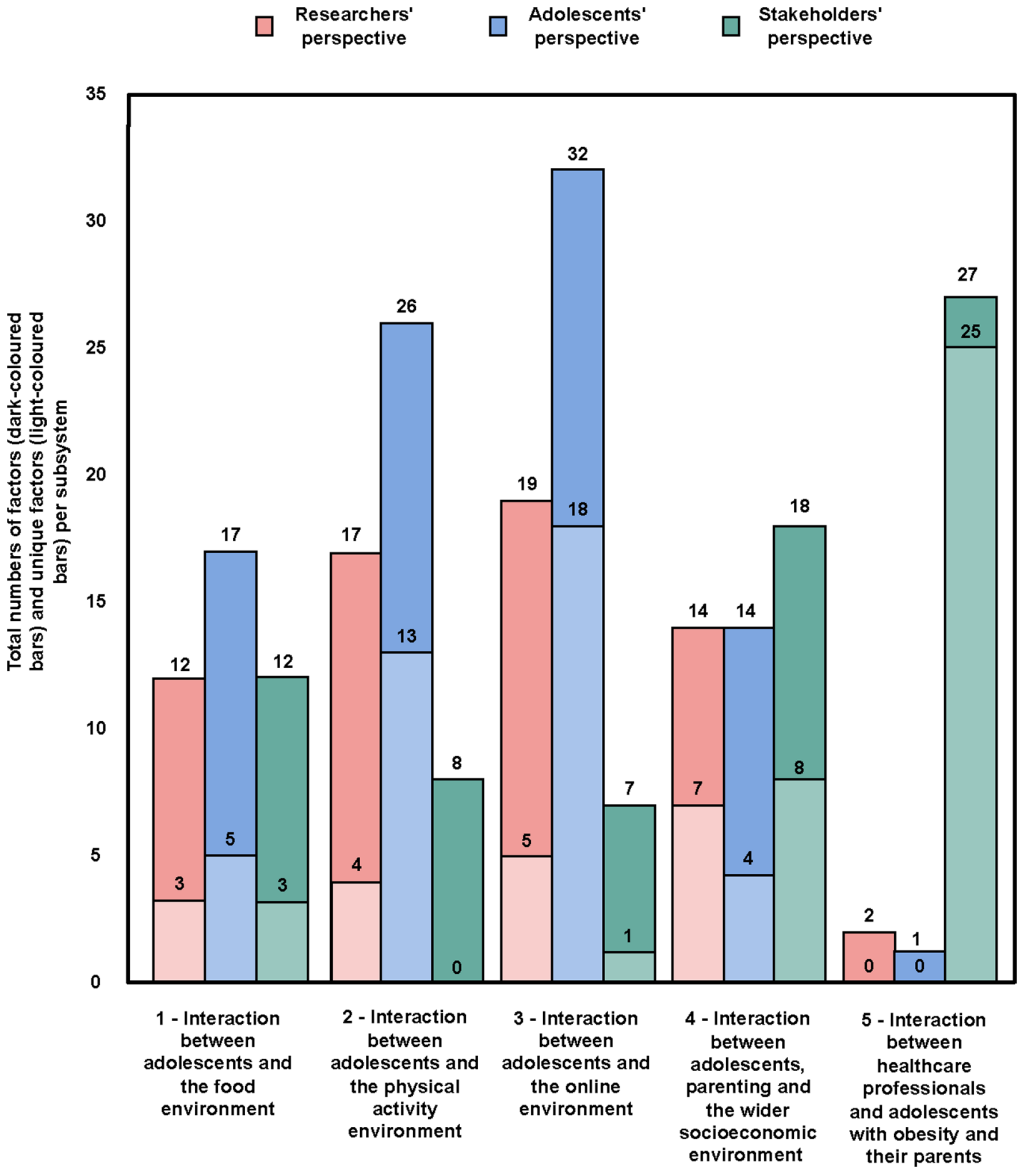
Total numbers of factors and unique factors from the perspectives of researchers, adolescents and stakeholders in subsystems 1–5.

In total, we identified 121 unique factors in the final systems map; 50 of these derived from the researchers’ perspective, 74 from the adolescents’ perspective and 54 from the stakeholders’ perspective (Figure 1). Due to overlap between the perspectives, the sum of the factors from all perspectives is greater than the total number of factors in the integrated system map. We also identified a total of 31 reinforcing feedback loops. Six different subsystems emerged (Figure 1). The total numbers of factors within each subsystem from the three perspectives, as well as the numbers of factors that were unique to a single perspective in each subsystem, are shown in Figure 2. Subsystem 6 is not shown in that figure, as the factors in that subsystem were embedded in the other five, as explained below. Identified factors, feedback loops, system structures and system goals will be discussed below separately for each of the six identified subsystems.

### 3.1. Subsystem 1: interaction between adolescents and the food environment

**Figure 3:**
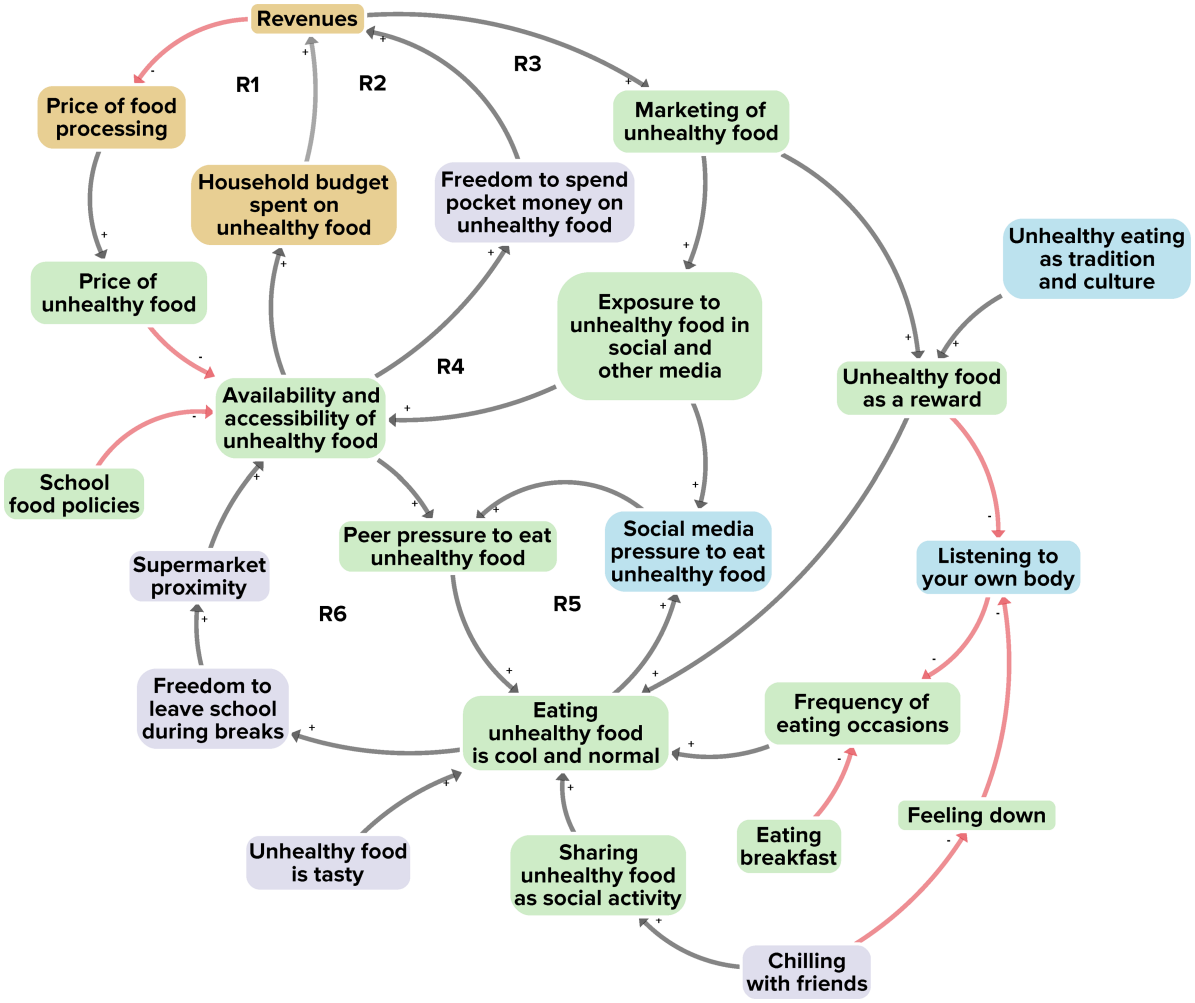
Subsystem 1: Interaction between adolescents and the food environment. Factors derived from the researchers’ perspective are shown in yellow, those from the adolescents’ perspective in purple, and those from the stakeholders’ perspective in blue. Factors present in at least two of the three perspectives are shown in green. Black arrows indicate positive polarity and red arrows indicate negative polarity in the causal relationship between factors.

Figure 3 illustrates the interaction between adolescents and the food environment. Out of a total of 23 factors, 12 were derived from the researchers’ perspective, 17 from the adolescents’ perspective and 12 from the stakeholders’ perspective. A total of 11 factors were unique to a single perspective. Six reinforcing feedback loops were identified as we integrated all perspectives (Figure 3, R1–R6).

The first two reinforcing feedback loops (R1, R2) relate to the relatively low price of unhealthy food, which makes unhealthy food more attractive and easily accessible. This boosts the demand for unhealthy food, which in turn allows food providers to maintain lower prices. The high demand for unhealthy food, in turn, reinforces the availability and accessibility of unhealthy food. The second two reinforcing feedback loops (R3, R4) reveal how this demand and supply chain of unhealthy food leads to high revenues, which can then be used for the marketing of such foods, thereby further reinforcing the availability and accessibility of unhealthy food.

Another feedback loop relates to the social norm that eating unhealthy food is cool and normal. In most larger Dutch towns and cities, a supermarket is found on almost every street corner. Visiting the supermarket with friends during school hours and buying unhealthy food together is seen by many adolescents as normal behaviour and as a fun and attractive social activity. This reinforces the social norm that eating unhealthy food is cool and normal (R6).

In addition to physical exposure, we found a feedback loop involving online exposure to unhealthy food. Adolescents typically spend a large amount of their time in online environments. Especially on social media platforms, peer pressure to buy and eat unhealthy food is commonly prevalent (for example when influencers advertise unhealthy foods) (R5). This further sustains the social norm that eating unhealthy food is cool and normal.

Taking together all 23 factors, their interconnections, and the six reinforcing feedback loops, we see a system structure revolving around the comparatively high availability, accessibility and affordability of unhealthy food. Such food may be preferred by adolescents not only because of the easy access, but also through the prevailing social norm that eating unhealthy food is cool and normal. This is further reinforced by marketing, social media and peer-group influence surrounding unhealthy foods. In terms of system goals, we observe that these factors belong to a larger system that focuses on profit maximisation, which can be achieved by selling as much food as possible – whereby unhealthy foods (heavily processed and with high energy density or high sugar, salt and fat content) are the more profitable option. For example, the stakeholders in our GMB workshops explained that local business owners prefer unhealthy over healthy foods, because the revenues are larger and the losses (as from food waste, logistics and cooling) are much lower.

### 3.2. Subsystem 2: interaction between adolescents and the physical activity environment

**Figure 4:**
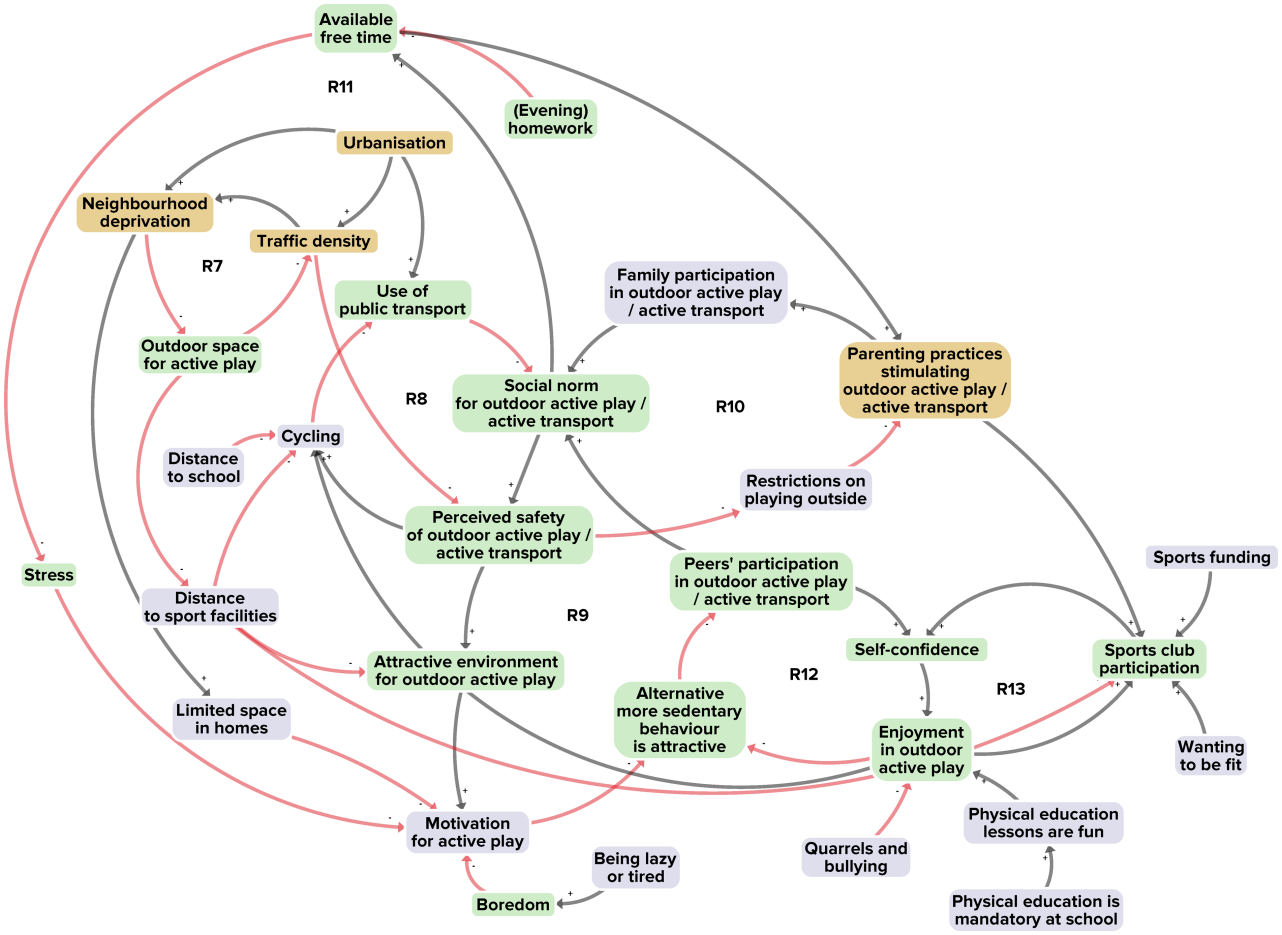
Subsystem 2: Interaction between adolescents and the physical activity environment. Factors derived from the researchers’ perspective are shown in yellow, and those from the adolescents’ perspective in purple. Factors present in at least two of the three perspectives are shown in green. Black arrows indicate positive polarity and red arrows indicate negative polarity in the causal relationship between factors.

Figure 4 illustrates the interaction between adolescents and the physical activity environment. A total of 31 factors emerged, of which 17 derived from the researchers’ perspective, 26 from the adolescents’ perspective and 8 from the stakeholders’ perspective. In total 17 of the factors were unique to a single perspective. Seven reinforcing feedback loops were identified in integrating the perspectives (Figure 4, R7–R13).

Reinforcing feedback loop R7 illustrates how urbanisation generally increases traffic density and neighbourhood deprivation, resulting in limited outdoor space for active play. The high demand for housing and businesses in cities like Amsterdam has prompted the building of sport facilities on the outskirts of neighbourhoods, thereby increasing the distance to the facilities; as a consequence, adolescents make less use of the facilities. A related factor is greater traffic density, which generally reduces the perceived safety of the physical activity environment. Adolescents then cycle less and make more use of public transport. This hampers sustainment of a healthy social norm of active outdoor play and active transportation (R8). The more the physical activity environment is perceived as unsafe, the more its attractiveness to adolescents declines, leading to lower participation by adolescents and their peers in active play and transport (R9). Also due to the perceived unsafety, parents will be less motivated to encourage habits of active play and transport, further weakening the healthy social norm (R10). In turn, once a social norm of active outdoor play and transport does not prevail, adolescents will be less encouraged to create free time for such activities, thus further reducing their motivation (R11). That may make alternative, more sedentary behaviours, such as screen use, more attractive (R11, R12) (thus linking with subsystem 3 below) and thereby make the physical activity environment all the less enjoyable (R12, R13).

Taking all 31 factors, their interconnections and their seven reinforcing feedback loops together, we see a system structure with dwindling availability of attractive, safe outdoor spaces for physical activity by adolescents. This undermines a healthy social norm of outdoor active play and active transportation. We note that this structure is part of a larger system goal that revolves around maximising utility for limited urban space by prioritising housing, business and motorised transport above outdoor space for active play.

### 3.3. Subsystem 3: interaction between adolescents and the online environment

**Figure 5:**
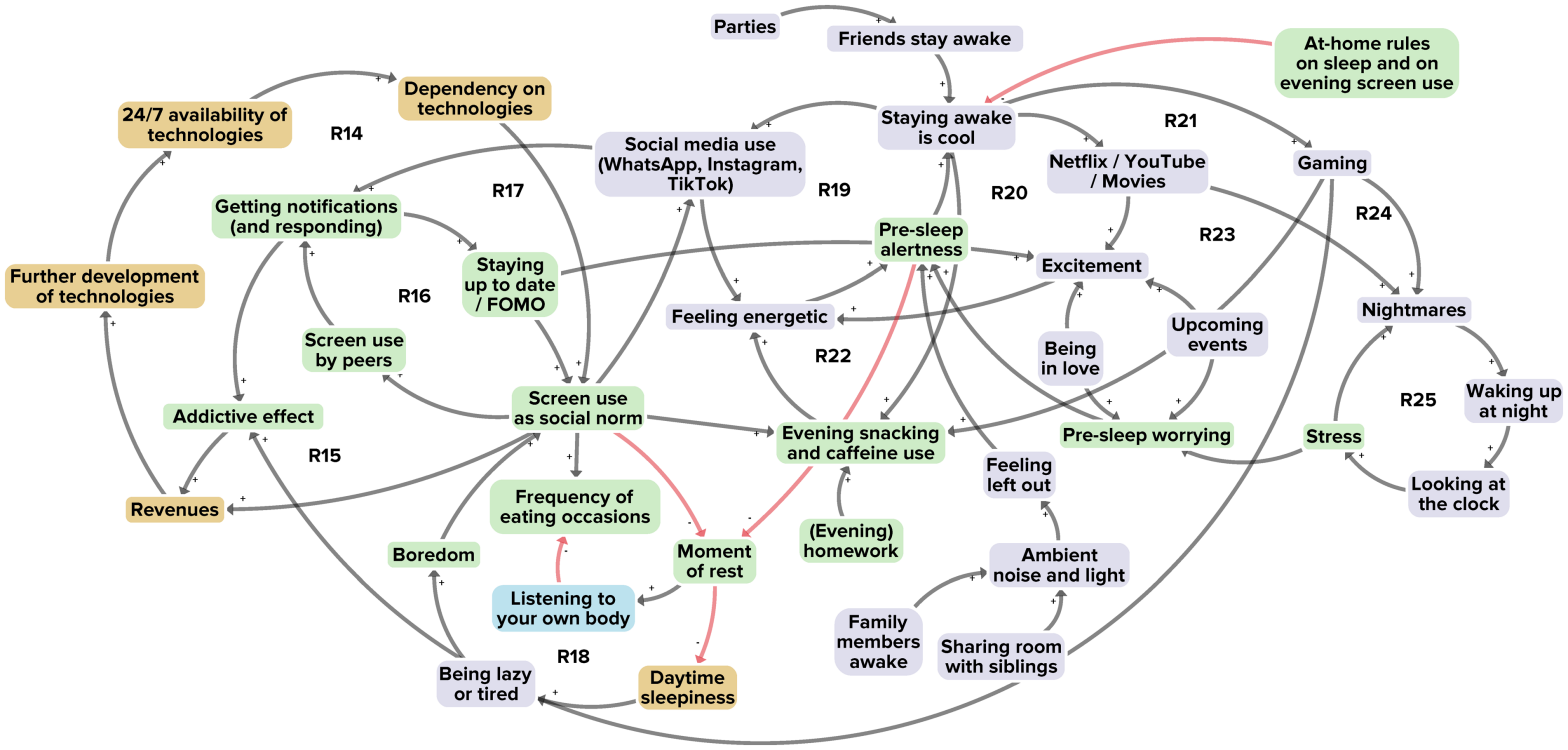
Subsystem 3: Interaction between adolescents and the online environment. Factors derived from the researchers’ perspective are shown in yellow, those from the adolescents’ perspective in purple, and those from the stakeholders’ perspective in blue. Factors present in at least two of the three perspectives are shown in green. Black arrows indicate positive polarity and red arrows indicate negative polarity in the causal relationship between factors.

Figure 5 illustrates the interaction between adolescents and the online environment. From a total of 38 factors, 19 derived from the researchers’ perspective, 32 from the adolescents’ perspective and 7 from the stakeholders’ perspective. A total 24 of the factors were unique to a single perspective. Twelve reinforcing feedback loops were identified in integrating the perspectives (Figure 5, R14–R25).

The first feedback loop (R14) relates to screen use as part of everyday life. Virtually all ordinary tasks of adolescents, including schoolwork, require using screens. This results in a society that is highly dependent on technology, and where the high demand and supply of new technologies further reinforce that dependency and help sustain the social norm of screen use as part of everyday life. The screen use norm is reinforced yet further by a fear among adolescents of missing out (FOMO) on what happens online; this induces an addictive effect of constantly wanting to be online (R15, R16). Social media use by adolescents plays herein an important role. The countless notifications received from WhatsApp, Instagram and TikTok further fuels adolescents’ curiosity to stay up to date, not to miss out, and hence to be perpetually online (R17). Adolescents’ high levels of screen use are not only common during the daytime; they also use screen devices before bedtime, adversely affecting sleep and reducing restful moments (R18). Social media use, watching Netflix, YouTube and movies, and gaming are activities frequently performed by adolescents in evening and nighttime hours (R19–R21). These reinforce a social norm that it is cool to stay awake (R19–R24). Screen use at night is often accompanied by snacking and caffeine use, giving adolescents an even greater sensation of energy, causing pre-sleep alertness and adversely affecting sleep and dietary behaviour (R21–R22). Furthermore, they often experience nightmares after gaming or watching horror movies, and this also affects sleep (R23–R25).

Taking together all 38 factors, their interconnections and twelve reinforcing feedback loops, we see a system structure revolving around 24/7 availability and accessibility of screens, whereby everyday life tasks are increasingly performed on screens. We observe that this screen use maximisation is part of a larger system whose goal is to maximise the profits obtained from technology use. For example, adolescents who like videogames generally follow their favourite gaming influencers on streaming channels. The more followers those influencers have, the more profits these can make through lucrative deals offered by private sector companies – such as for advertising unhealthy food in their videos – and the more profits those companies eventually make.

### 3.4. Subsystem 4: interaction between adolescents, parenting and the wider socioeconomic environment

**Figure 6:**
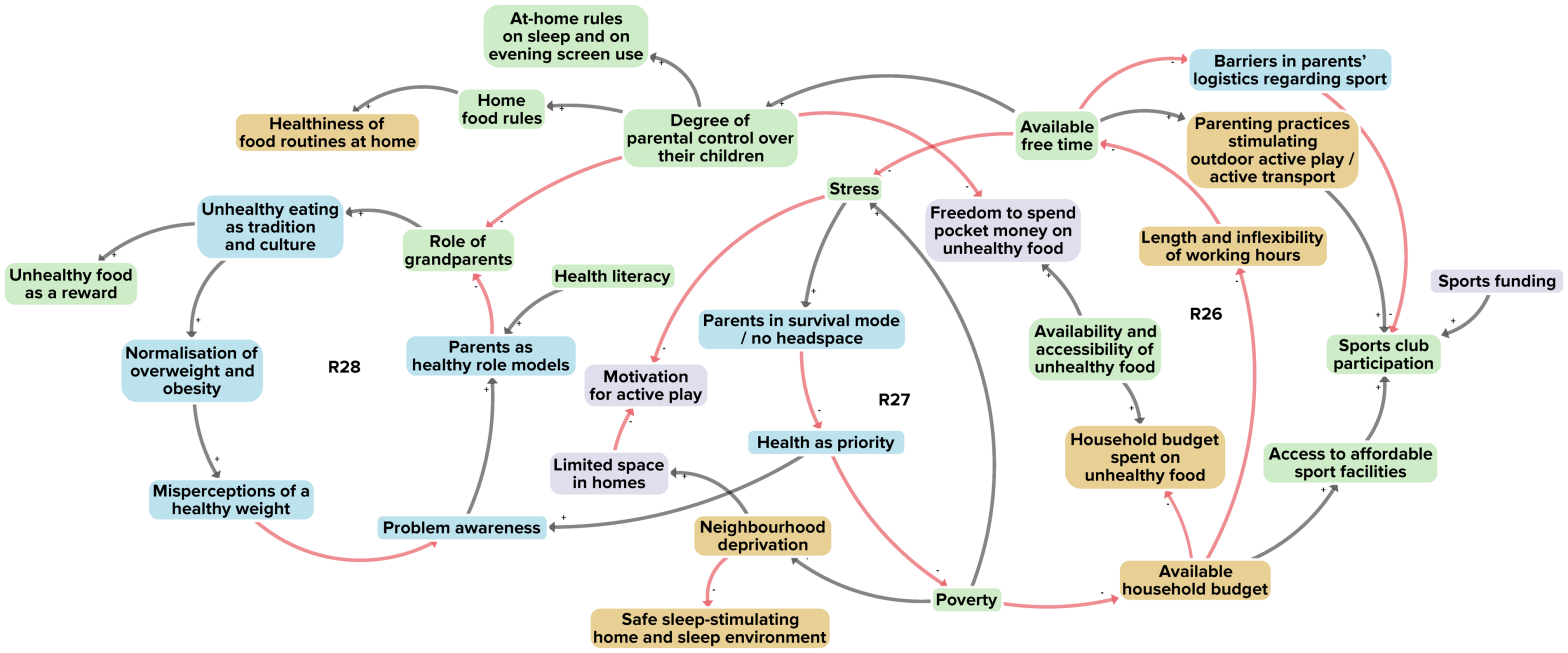
Subsystem 4: Interaction between adolescents, parenting and the wider socioeconomic environment. Factors derived from the researchers’ perspective are shown in yellow, those from the adolescents’ perspective in purple, and those from the stakeholders’ perspective in blue. Factors present in at least two of the three perspectives are shown in green. Black arrows indicate positive polarity and red arrows indicate negative polarity in the causal relationship between factors.

Figure 6 illustrates the interaction between adolescents, parenting and the wider socioeconomic environment. In a total of 31 factors, 14 derived from the researchers’ perspective, 14 from the adolescents’ perspective and 18 from the stakeholders’ perspective. A total of 19 of the factors were unique to a single perspective. Three reinforcing feedback loops were identified in integrating the perspectives (Figure 6, R26–R28).

The first feedback loop (R26) relates to a large number of households in our research community living in relative poverty, where parents typically have long, inflexible working hours and hence limited free time and higher stress levels. This, in turn, may put parents in a ‘survival mode’, leaving limited headspace for matters such as preparing healthy meals. Parents find themselves in a vicious circle as financial problems accumulate; that triggers even more stress, as they often need to solve such multiple problems in a short time span (R27).

With such financial problems occupying parents’ headspace, they often pay less attention to their children’s health behaviours. As parents have less time for their children, grandparents may play a greater role in the upbringing of adolescents (R28). In our research community, a large percentage of such grandparents come from cultures where unhealthy eating may be seen as tradition and culture, for example when guests are welcomed with an abundance of food, usually unhealthy.

In combination with the parents’ limited headspace, their transition to their new role as coaches or mentors of young adolescents, rather than childrearers of younger children, commonly makes it difficult for them to set, monitor and enforce rules regarding sleep, dietary behaviour, screen behaviour and physical activity.

Taking together all 31 factors, their interconnections and three reinforcing feedback loops, we see a system structure that revolves around parents’ limited capabilities to stimulate healthy behaviours, in particular in ethnically diverse groups of lower socioeconomic status. Parents are subject to competing demands and stressors, possibly relating to financial worries, long working hours, general uncertainty, and traditional cultural roles and patterns. We note that this is part of a larger system whose goals prescribe individual responsibility while compelling parents to prioritise household livelihood security at the expense of stimulating healthy behaviours.

### 3.5. Subsystem 5: interaction between healthcare professionals and adolescents with obesity and their parents

**Figure 7:**
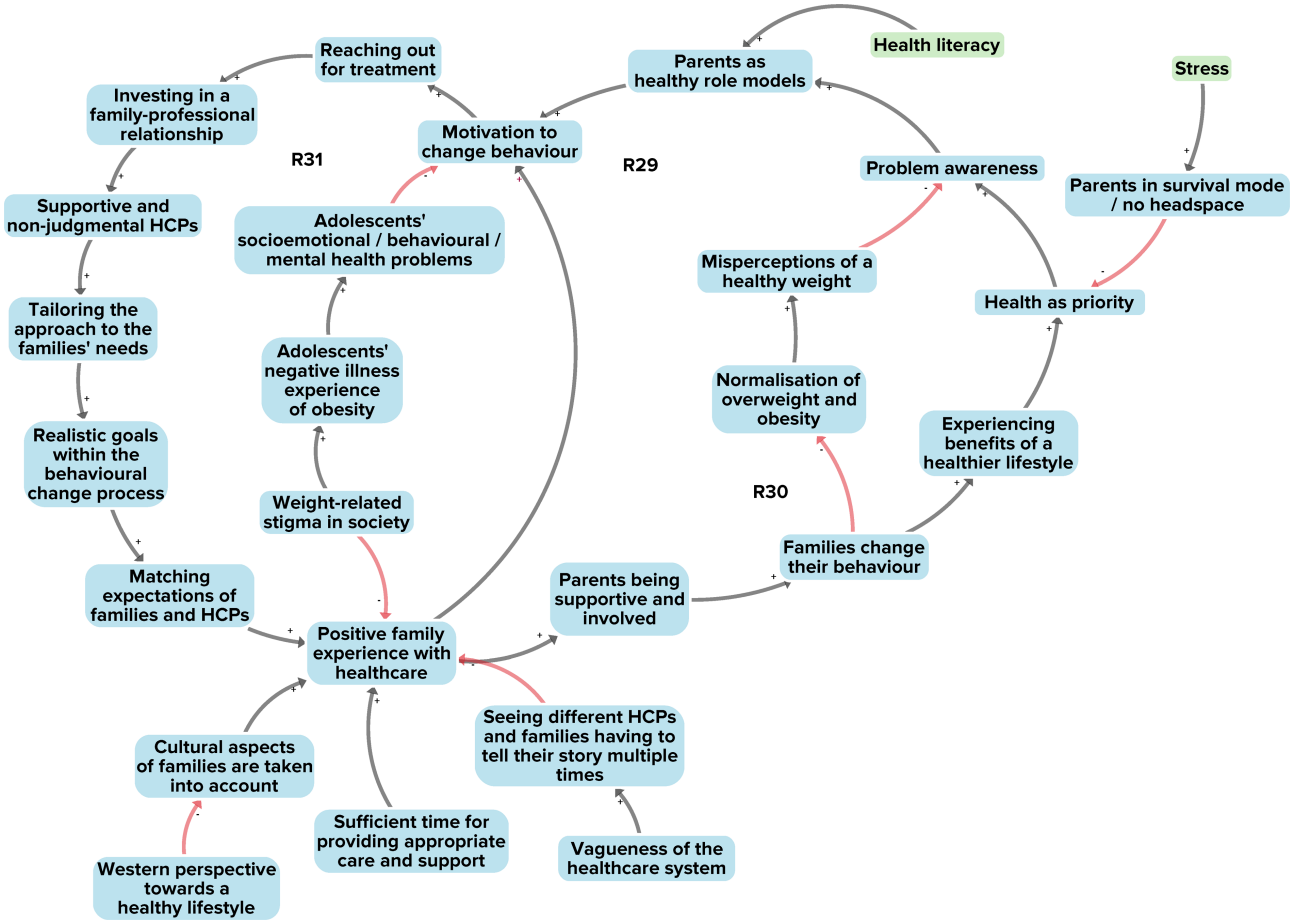
Subsystem 5: Interaction between families and healthcare. Factors derived from the stakeholders’ perspective are shown in blue. Factors present in at least two of the three perspectives are shown in green. Black arrows indicate positive polarity and red arrows indicate negative polarity in the causal relationship between factors.

Figure 7 illustrates the interaction between healthcare professionals (HCPs) and adolescents with obesity and their parents. From a total of 27 factors, 2 factors derived from the researchers’ perspective, 1 from the adolescents’ perspective and 27 from the stakeholders’ perspective. A total of 25 of these factors were unique to a single perspective, that of the stakeholders. The reason for the comparatively large number of factors in the stakeholder perspective is that ‘healthcare’ was not included nor discussed as a potential subsystem in the researchers’ and adolescents’ original data, but only in the stakeholder data. Moreover, in contrast to the other identified subsystems, the healthcare subsystem data relates specifically to adolescents *with obesity* in a healthcare setting or context, rather than to the general population. Three reinforcing feedback loops were identified (R29–R31).

All three of the reinforcing feedback loops were linked to a single feedback loop outlined in subsystem 4 involving the interaction between adolescents, parenting and the wider socioeconomic environment (Figure 6, R26). It showed that poorer families in our research community were often in survival mode, with limited headspace to think about health-related behaviours. This feedback loop feeds into the factors of ‘low general priority for health’ and ‘limited awareness of a health problem’ (in this case, overweight) (R29, R30). From the perspective of HCPs, this results in families showing little motivation to change unhealthy behaviours; this could lead to normalisation of overweight and obesity and to misperceptions of what constitutes a healthy weight (R30).

The three reinforcing feedback loops further show that a number of factors are important to ensure that families have a positive healthcare experience. These include investing in a family–professional relationship, offering a treatment approach tailored to a family’s needs, and managing treatment expectations between families and HCPs (R31). The interviews with HCPs revealed that achieving these aims is not automatically assured. One challenging situation may arise when HCPs regard a healthy lifestyle from a Western European perspective, hence not sufficiently taking the cultural diversity of families into account. Culture serves here as an example of underlying factors related to obesity that may not be readily observable to HCPs but may nevertheless contribute to the problem.

Taking together all the 27 factors, their interconnections and the three feedback loops, we see a subsystem where many conditions, such as a family–professional relationship and a tailored approach to a family’s needs, must be met if adolescents with obesity and their parents are to modify and sustain health behaviours. The interviews with HCPs revealed that these conditions have not yet been fully achieved in the healthcare system, for reasons such as insufficient time for appropriate care and support and insufficient consideration of families’ cultural aspects by HCPs. This results in a system that treats obesity mainly as an isolated medical problem, with little attention for the social and cultural contexts that affect problem management by adolescents and parents.

### 3.6. Subsystem 6: transition from childhood to adolescence

In analysing the sixth subsystem, we took a slightly different approach as compared to previous subsystems. The reason is that the factors relating to the child-to-adolescent transition are embedded within the various other subsystems (Figure 1), rather than forming feedback loops that are unique to this subsystem itself. Subsystem 6 therefore tightly interacts with the five subsystems previously discussed.

We noted that, during this transition period, adolescents are extra susceptible to the influence of the system they are a part of. Such susceptibility may manifest itself in a display of obesity-related behaviours. During the transition, adolescents generally increase their consumption of unhealthy foods (subsystem 1), decrease their levels of physical activity (subsystem 2) and increase their sleep-affecting screen time (subsystem 3). We identified three principal factors that foster susceptibility to systemic influence. The first relates to the adolescent urge for freedom. Greater autonomy and independence enables them, for example, to purchase unhealthy food from easy accessible environments (such as supermarkets). The second factor reflects the adolescent desire to be part of and accepted by a group, making them particularly vulnerable to peer pressure and to influences from social media. The third factor involves seeking instant gratification. It is more gratifying for adolescents to spend long hours gaming with their friends and ‘enjoying the moment’ (subsystem 3) than to force themselves to be physically active because that would be good for their health (subsystem 2). Long-term health benefits are not typically prioritised by adolescents during this transition period; and parents, who could help curb unhealthy habits, may experience diminished influence on their children (subsystems 4 and 5). During the transition from childhood to adolescence, parents shift from a childrearing role to more of a coaching or mentoring role. The new role can make it difficult for parents to set, monitor and enforce rules about healthy behaviours (subsystem 4).

We conclude that the wider system goal here is linked to biological and psychosocial mechanisms, which include increased autonomy and independence, susceptibility to peer pressure and social media exposure, and gratification-seeking – factors that make adolescents specifically susceptible to an environment that fosters obesity-related behaviours. Adolescents report, for instance, that they are continuously exposed to a multitude of unhealthy food advertisements and providers in their close surroundings. This may not only trigger a craving for unhealthy food, but it may also constrain them from escaping that environment to seek healthier foods and activities.

## 4. Discussion

This study sought to identify and understand the underlying system dynamics that drive obesity-related behaviours in adolescents. We developed a CLD with a multi-actor perspective and subsequently performed systems-based analysis to understand the pre-existing system in terms of both system structure and function. The focus was on adolescents aged 10 to 14 in an urban setting. The resulting CLD contains 121 unique factors, 31 feedback loops and 6 subsystems (revealing system structure) with their corresponding system goals (revealing system function).

The first subsystem reveals the interaction between adolescents and the food environment. The system goal is *profit maximisation*, which can be achieved by selling as much food as possible, with the more profitable option being unhealthy foods (heavily processed, high energy density, high in sugar, salt or fat). Subsystem 2 shows the interaction between adolescents and the physical activity environment, whereby the system goal is *utility maximisation* for limited urban space, with housing, business and motorised transport prioritised above outdoor space for active play. Subsystem 3 focuses on the interaction between adolescents and the online environment, with a system goal of *profit maximisation from technology use*. Subsystem 4 shows the interaction between adolescents, parenting and the wider socioeconomic environment; system goals prescribe *individual responsibility*, which may compel parents to prioritise household livelihood security at the expense of stimulating healthy behaviours. Subsystem 5 highlights interaction between healthcare professionals and families, with a system goal under which obesity is *treated as an isolated medical problem*, with insufficient attention to social and cultural contexts that may hinder adolescents and their parents in managing the problem. Subsystem 6 relates to the dynamics of the child-to-adolescent transition, which can also be seen as an element in each of the other five subsystems; here the system goal relates to *biological and psychosocial mechanisms* – increased autonomy and independence, susceptibility to peer pressure and social media exposure, seeking instant gratification – which make adolescents particularly vulnerable to an environment that fosters obesity-related behaviours.

### 4.1. Findings relating to system structure

The CLD presented in this study shows the combined perspectives of academic researchers, adolescents and stakeholders. Overall, adolescents contributed the most factors to the CLD (74/121), followed by stakeholders (54/121) and researchers (50/121). That finding applied both to unique factors and to factors deriving from multiple perspectives, and it underlines the importance of including multiple perspectives. For example, in subsystem 3 (interaction between adolescents and the online environment), the researcher and stakeholder perspectives highlighted the social norm around screen use as a key mechanism in this subsystem. However, only after we included the adolescents’ perspective did it become apparent what this mechanism actually meant to adolescents – that screen use in the form of social media, gaming and movie-watching serves to sustain a social norm that it is cool to stay awake at night.

We further explored that finding by highlighting the factors in the CLD separately for each perspective (Supplementary Figures S1–S3); this reveals that important information on the system structure is lost in each separate CLD. For example, looking at the feedback loops for each single perspective, we found 7 loops for the academic researchers, 12 loops for the adolescents and 5 loops for the stakeholders, whereas integrating the perspectives resulted in 31 reinforcing feedback loops. Generally speaking, the researchers’ and stakeholders’ perspectives contributed to the exposure of the system structure, of *how a specific environment works*, whereas integration of the adolescents’ perspective revealed *the ways in which adolescents interact* with this environment. For example, from the researcher perspective we learned that screen use as a social norm is sustained by an environment that reinforces supply and demand for technological devices. The adolescent perspective then showed how that social norm is *further* sustained in activities like purchasing the latest video gaming devices in the market and using them as instruments of peer interaction in the online world. Previous studies have likewise underlined the importance of including multiple perspectives to obtain a fuller understanding of a system (16). In a study by McGlashan and colleagues (29), factors present in a Foresight map (9) were compared with factors present in a map developed by community stakeholders (11). This showed that the largest proportion of factors in the Foresight map focused on the physiology cluster (23%), whereas social psychology was the largest cluster in the community stakeholders’ map (38%), with a mere 2% of factors focused on physiology.

### 4.2. Findings relating to system function

Whilst analysis of system structure in terms of system factors and feedback loops provides important information about a system, it does not yet provide insights into the deeper system dynamics (system goals). The latter can be referred to as *system function*, and it is crucial for understanding, and subsequently changing, the system as a whole.

First, our analysis of the system as a whole revealed that the system primarily contains reinforcing feedback loops encouraging obesity-related behaviours, without balancing feedback loops discouraging the behaviours. While this finding can partly be explained by the methods we used (with a focus on obesity-related behaviours), it does show a system geared to reinforcing obesity-related behaviours. One subsystem that could potentially serve as a balancing loop is the healthcare system (subsystem 5). In practise, however, the conditions for good obesity care – where social and cultural contexts would form an integral part of the treatment of adolescents with obesity – are not yet being fully satisfied. Moreover, even if such conditions were to be met, healthcare can, at best, provide an answer to only part of the system – by helping those who are already overweight. It cannot prevent obesity-related behaviours from occurring in the first place.

Second, when we examine the functioning of this system in terms of emergent properties at the individual level, we observe a system that gears people towards instant gratification in terms of social media likes, tasty food, belonging to a group and other pleasures. Such gratification is specifically important for young adolescents in the transition from primary to secondary school, in that they are suddenly exposed to greater autonomy, with growing peer-group influence and diminishing parental supervision (30–33). At the same time, parents themselves struggle with this new phase, in particular with regard to a lack of parenting skills surrounding mobile phone and social media use (34–37).

Third, when looking at the emergent properties of the system at a macro level, we see that the system function for multiple, but not all, subsystems revolves around the goal of maximising short- or longer-term economic growth in the paradigm of a market-driven economy. Private-sector companies are known to use strategies that promote specific products and choices that are detrimental to health (38). Specific examples of the conflicting system goals from public health and commercial perspectives can also be found in the growing commercial determinants of health literature. This points up the fundamental conflict between imperative shareholder value maximisation and population health (38). In agreement with previous research, our analysis has shown that young people in the child-to-adolescent transition period are particularly susceptible to the marketing and production strategies of commercial companies. That derives from adolescents’ peer influences, their immature cognitive and emotional development, and their high exposure to unhealthy foods in their physical and online environments (39–41).

While it is obviously highly challenging to influence macro system functions, it is important to understand the system in which we are operating, and to be aware that any public health intervention aiming to change the system will have to work within (or probably against) that system. Having such system knowledge will likely result in the development of different types of interventions and programmes (19, 28). For example, the social marketing literature shows us how instruments from traditional marketing (product, price, promotion, place) can be used to ‘sell’ healthier alternatives. However, even though such a social marketing approach may benefit individuals, groups or societies as a whole (42–44), it still does not address the system goals. Placing cartoon characters on fruit, for example, will not address the marketing mechanisms that make unhealthy food attractive and profitable. The emerging field of systems social marketing indeed emphasises the need to adopt a more holistic or systems mode of operandi (45). A more systemic alternative would include a full understanding and consideration of the adolescents’ perspective in efforts to promote a particular health outcome. For example, adolescents indicated to us that they find their physical environment unattractive and boring, as it is designed mainly for young children. If adolescents were to have a voice in the design of outdoor spaces, they might make more use of such spaces and increase their levels of physical activity.

### 4.3. Strengths and limitations

To the best of our knowledge, this is the first study that combines a multi-actor perspective with a system-based analysis in order to understand the dynamics of obesity-related behaviours. A limitation of our study is that, while we combined different perspectives from the original data sources in our aggregated CLD, the system-based analysis and interpretation was performed only from the academic perspective. Ideally, one would feed the final results back to the adolescents and the stakeholders to make sure our interpretation agrees with their perceptions of the system; or one might even involve adolescents and stakeholders in the analytic process. However, such system analysis without proper guidance might have been challenging for the groups involved here, in particular because not all subsystems identified in our study (such as subsystem 5) were discussed in the original single-perspective data. Nevertheless, authors that were involved in the original data collection on the various perspectives were also involved in the system analysis, and we checked our interpretations against their original data.

Another limitation may be that, although systems are dynamic, the figurations of the system as presented in our study may seem static. Our results should therefore be interpreted as the understanding we developed from snapshots of the pre-existing system, while still bearing in mind that system understanding is a progressive process. The identified subsystems and the concurrent system goals highlighted in our study can serve as a basis for locating points to intervene in the system, also known as leverage points (1). Foster-Fishman and colleagues refer to this step as the final information needed to successfully develop and implement interventions that can alter the status quo of targeted systems (16). In the LIKE programme, we indeed seek to use the insights obtained from the present study as a basis to find leverage points and develop actions to help change the system into a healthier system for adolescents.

Finally, it is important to point out that the uncovered underlying system dynamics described in this study refer to those dynamics found to be relevant to our target group (10- to 14- year-old adolescents) in the context of a Western urban setting. The observed dynamics are a result of our methods which relied on academic experts’ perspective and interpretation, and adolescents’ and stakeholders’ perspectives. For that reason, the resulting pre-existing system CLD of obesity-related behaviours does not present evidence for the exact working of the system dynamics but should rather be interpreted as one piece of a bigger puzzle. Indeed, we did not intend to develop a full conceptual model of childhood overweight and obesity, but one that focused on our target group and setting. However, the types of dynamics (feedback loops, subsystems, and goals) identified in this study are also relevant in other contexts. For example, subsystems that have as goal economic profit.

## 5. Conclusion

Our paper has confirmed the relevance of combining multiple perspectives in gaining system understanding of obesity-related behaviours. The researchers’ and stakeholders’ perspectives contributed in particular to an understanding of how the system structure of the obesogenic environment works. Integrating the adolescents’ perspective enriched the insights on how adolescents interact with that environment. The system analysis revealed that the system in which adolescents live is composed of multiple subsystems that interact with one another and whose goals serve to reinforce obesity-related behaviours over time. Multiple subsystems operate within a paradigm which, on the individual level, maximises short-term gratification; this is intensified by factors such as the urge for freedom that characterise the transition from childhood to adolescence. On the macro level, the paradigm maximises economic growth. Understanding such types of system drivers is crucial for the development of future interventions.

## Data availability statement

The original contributions presented in the study are included in the article/Supplementary material, further inquiries can be directed to the corresponding author.

## Ethics statement

The studies involving human participants were reviewed and approved by the institutional medical ethics committee of Amsterdam UMC, Location VUMC (2018.234). Written informed consent to participate in this study was provided by participants or by the participants’ legal guardian/next of kin.

## Author contributions

ALP, KS, and WW: conception and design of the work and writing original draft. KS and WW: supervision. KS, CD, CR, TA, MC, and SK: funding acquisition. ALP, KS, HE, EvdE, TA, CD, CR, RH, VB, MC, SK, and WW: interpretation and critically reviewing manuscript. All authors contributed to the article and approved the submitted version.

## Funding

This work was supported by a grant from the Netherlands Cardiovascular Research Initiative: An initiative with support of the Dutch Heart Foundation, ZonMw, CVON2016-07 LIKE.

## Conflict of interest

The authors declare that the research was conducted in the absence of any commercial or financial relationships that could be construed as a potential conflict of interest.

## Publisher’s note

All claims expressed in this article are solely those of the authors and do not necessarily represent those of their affiliated organizations, or those of the publisher, the editors and the reviewers. Any product that may be evaluated in this article, or claim that may be made by its manufacturer, is not guaranteed or endorsed by the publisher.

## Abbreviations

CLD: Causal loop diagram
GMB: Group model building
LIKE: Lifestyle Innovations Based on Youth Knowledge and Experience programme
HCPs: Healthcare professionals
